# Genomic and ecological attributes of marine bacteriophages encoding bacterial virulence genes

**DOI:** 10.1186/s12864-020-6523-2

**Published:** 2020-02-05

**Authors:** Cynthia B. Silveira, Felipe H. Coutinho, Giselle S. Cavalcanti, Sean Benler, Michael P. Doane, Elizabeth A. Dinsdale, Robert A. Edwards, Ronaldo B. Francini-Filho, Cristiane C. Thompson, Antoni Luque, Forest L. Rohwer, Fabiano Thompson

**Affiliations:** 1 Department of Biology, San Diego State University, 5500 Campanile Dr, San Diego, CA 92182 USA; 2 Viral Information Institute, San Diego State University, 5500 Campanile Dr, San Diego, CA 92182 USA; 3 Department of Biology, University of Miami, 1301 Memorial Dr., Coral Gables, FL 33146 USA; 4 Departamento de Producción Vegetal y Microbiología, Universidad Miguel Hernández, Apartado 18, 03550 San Juan de Alicante, Spain; 5 Sydney Institute of Marine Science, 19 Chowder Bay Rd, Mosman, NSW 2088 Australia; 6 Centro de Biologia Marinha, Universidade de São Paulo, Rodovia Manoel Hypólito do Rego, Km 131,50, São Sebastião, SP 11600-000 Brazil; 7 Instituto de Biologia, Universidade Federal do Rio de Janeiro, Av. Carlos Chagas Filho, 373, Rio de Janeiro, RJ 21941- 599 Brazil; 8 Department of Mathematics and Statistics, San Diego State University, 5500 Campanile Dr, San Diego, CA 92182 USA; 9 Computational Science Research Center, San Diego State University, 5500 Campanile Dr, San Diego, CA 92182 USA; 10 SAGE/COPPE, Universidade Federal do Rio de Janeiro, Av. Carlos Chagas Filho, 373, Rio de Janeiro, RJ 21941- 599 Brazil

**Keywords:** Marine phage, Virulence genes, Lysogeny, Virome, Bacterial pathogenicity

## Abstract

**Background:**

Bacteriophages encode genes that modify bacterial functions during infection. The acquisition of phage-encoded virulence genes is a major mechanism for the rise of bacterial pathogens. In coral reefs, high bacterial density and lysogeny has been proposed to exacerbate reef decline through the transfer of phage-encoded virulence genes. However, the functions and distribution of these genes in phage virions on the reef remain unknown.

**Results:**

Here, over 28,000 assembled viral genomes from the free viral community in Atlantic and Pacific Ocean coral reefs were queried against a curated database of virulence genes. The diversity of virulence genes encoded in the viral genomes was tested for relationships with host taxonomy and bacterial density in the environment. These analyses showed that bacterial density predicted the profile of virulence genes encoded by phages. The Shannon diversity of virulence-encoding phages was negatively related with bacterial density, leading to dominance of fewer genes at high bacterial abundances. A statistical learning analysis showed that reefs with high microbial density were enriched in viruses encoding genes enabling bacterial recognition and invasion of metazoan epithelium. Over 60% of phages could not have their hosts identified due to limitations of host prediction tools; for those which hosts were identified, host taxonomy was not an indicator of the presence of virulence genes.

**Conclusions:**

This study described bacterial virulence factors encoded in the genomes of bacteriophages at the community level. The results showed that the increase in microbial densities that occurs during coral reef degradation is associated with a change in the genomic repertoire of bacteriophages, specifically in the diversity and distribution of bacterial virulence genes. This suggests that phages are implicated in the rise of pathogens in disturbed marine ecosystems.

## Background

With a total estimated abundance of 10^31^ particles, bacteriophages are the most abundant biological entities on Earth, and represent an untapped wealth of genetic information [1]. Bacteriophage genomes undergo frequent lateral gene transfers, and phage-encoded genes can be shared with microbial hosts and fixated under selective pressure [2–4]. Viral genome size is constrained by the capsid volume and mutation rates, resulting in condensed genomes with frequent overlapping open reading frames [5–7]. Thus, the ubiquitous presence of genes encoding bacterial cellular functions in viral particles suggests that most of these genes bring adaptive advantage to the viruses [3, 4]. Yet, the environmental drivers of phage genomic composition just recently started to be described [3, 8, 9].

The expression of phage genes during infection confers new functions and modulates existing host functions [10–12]. Bacterial virulence genes are often carried by temperate phages, and lysogenic conversion (the change in bacterial phenotype as a result of phage integration) is a major mechanism for the emergence of pathogens [13]. The genus *Vibrio* includes several examples of virulence acquisition through phage integration, including the human pathogen *Vibrio cholerae* [14]. The CTX toxin in *V. cholera* is a canonical example of phage-encoded pathogenicity through the direct acquisition of a toxicity function, but also through the regulation of the global bacterial transcriptome increasing the pathogen’s fitness in the animal-associated environment [15]. Prophages inserted in the genome of the coral pathogen *Vibrio corallilyticus* show high nucleotide sequence identity and similar gene organization with virulence gene-encoding *V. cholerae* phages, suggesting that lysogenic conversion cause coral disease [16, 17]. Altogether, these studies suggest that phage-mediated bacterial virulence contribute to pathogenicity in many marine diseases. However, a community-level analysis of phage-encoded virulence genes in marine environments is still missing.

The rise of fleshy macroalgae (coral competitors) in degraded coral reefs fuels microbialization, the increase in bacterial biomass and energetic demands [18–21]. High bacterial densities are accompanied by increases in the abundance of temperate phages encoding bacterial virulence genes and the frequency of lysogenic infections, a dynamic named *Piggyback-the-Winner* (PtW) [20, 22–24]. During microbialization, the bacterial community also becomes dominated by super-heterotrophs, including Gammaproteobacteria and Bacteroidetes [13, 25–28]. If the phage-encoded virulence genes bring niche expansion and competitive advantage to the bacterial hosts during microbialization, the selection of these genes will lead to genomic adaptation observed as changes in the gene functions and relative abundances. These changes should be correlated with both bacterial densities and phage host taxonomy.

A meta-analysis of virome-assembled viral genomic sequences from coral reef boundary layers (water overlaying corals) in the Atlantic and Pacific was employed here to test these predictions. Phage-encoded virulence gene profiles were significantly predicted by microbial densities. However, there was only marginal evidence for a role of host taxonomy in virulence gene distribution. These findings indicate that phages represent a reservoir of bacterial virulence factors in marine environments that contributes to the rise of pathogens during microbialization.

## Results

### Viral community structure and diversity

**Fig. 1:**
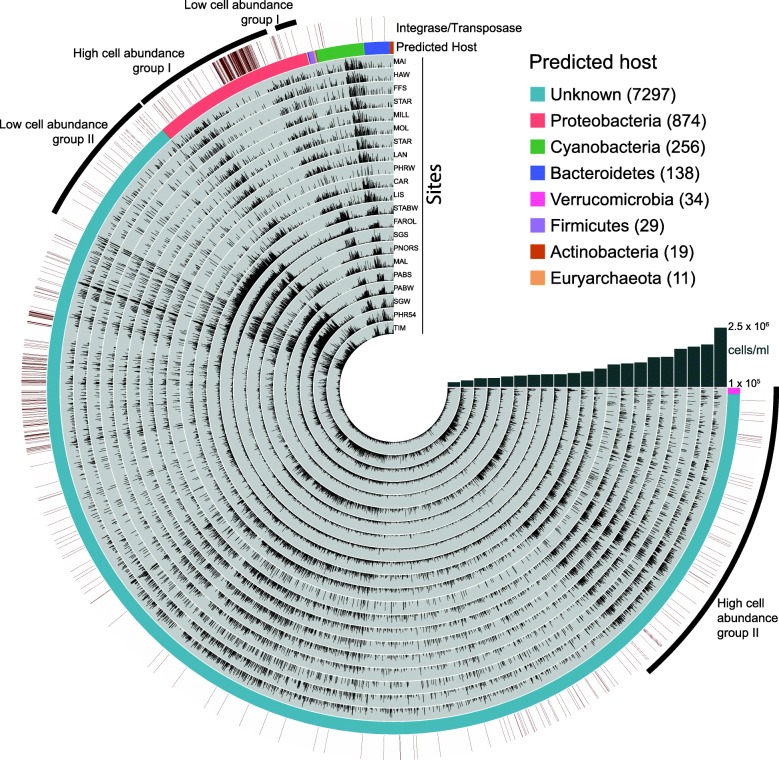
Relative abundances of Viral Genome Sequences (VGS). VGS are grouped by predicted host and viromes are ordered by the total microbial abundance in the reef site where they were collected. The inner grey rings show the abundance of each viral genomic sequence (VGS) in the viromes. The intermediary colored ring indicates predicted host (color legend located in the top right side of the figure). The outer ring indicates the presence of integrase genes identified through tBLASTx comparison with integrases and transposases from the viral RefSeq. Outer brackets indicate contigs infecting Proteobacteria and unknown hosts that increased in relative abundances at high or low cell abundance environments

A total of 28,483 Viral Genomic Sequences (VGS) representing virome-assembled viral genomic sequences (herein referred to as viral genomes) composed the viral community in the coral reefs analyzed here, recruiting 49.8 ± 2.2% (mean ± SD) of virome reads per site (Fig. 1). The host of most of these viruses could not be predicted (24,297 genomes recruiting 64.5% of all hits, on average across all samples), followed by viruses predicted to infect Proteobacteria (2281 genomes with 21.8% of hits), Cyanobacteria (1084 genomes with 11.5% of hits), and others (821 genomes with 1.98% of hits). The phage community structure, defined by the relative abundances of phage genomes, was significantly predicted by microbial densities at the reef site (high and low cell abundance groups in Fig. 1 and non-Metric Multidimensional Scaling analysis in Additional file 1: Figure S1, permutational linear model *p* = 0.001, pseudo-F_1,19_ = 5.42 using the relative abundances of genomes in each virome as response and Log_10_ of cell abundance as predictor variable).

**Fig. 2:**
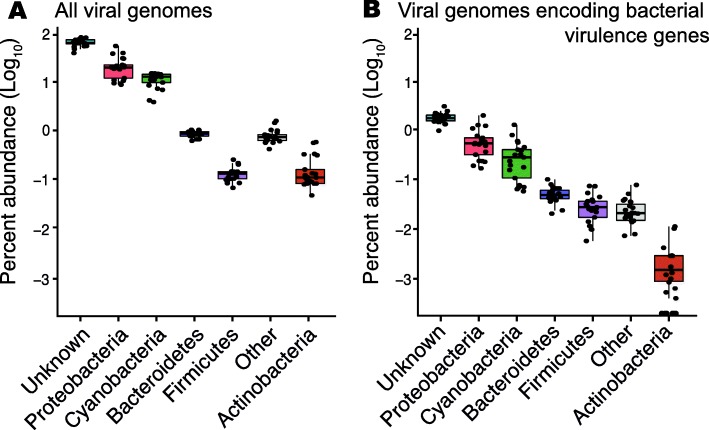
Predicted hosts of virulence-encoding viruses. Relative abundance (Log_10_) of viral genomes grouped by predicted host. **a** Abundance of genomes encoding bacterial virulence genes and **b** abundance of all viral genomes in the coral reef communities. In both cases, most viruses infect unidentified hosts, followed by Proteobacteria and Cyanobacteria

The rank-abundance curve built with mean relative abundances of viral genomes across all 21 viromes indicated that the community was highly diverse (Fig. 2 and Additional file 1: Figure S1). Only two members displayed abundances above 1%. Site-specific diversity was 7.47 ± 0.19 for Shannon index (mean ± SE), 14,589 ± 1481 for species abundance, and 0.79 ± 0.01 for evenness (Additional file 1: Table S1 shows diversity indexes for each site). The Shannon diversity had a negative relationship with microbial density in each site (linear regression *p* = 0.04, R^2^ = 0.18, Additional file 1: Figure S3A). Species abundance estimates were also negatively related with microbial abundances, having a steeper and significant negative slope (linear regression *p* = 4.53e-05, R^2^ = 0.59, Additional file 1: Figure S3B). The steep decrease in viral species abundance with increasing microbial abundance led to no change in community evenness despite the decrease in Shannon diversity (linear regression between evenness and microbial abundance *p* = 0.63).

### Virulence gene profile

A total of 1149 viral genomes accounting for 2 to 4% of the viral community encoded at least one bacterial virulence gene (Fig. 1 and Additional file 1: Figure S1). There was a trend for higher frequency and number of copies of virulence genes in low abundance viruses, although the relationship was not significant (Additional file 1: Figure S1, inlet; linear regression *p* = 0.08, a = 0.14). Most of the virulence-encoding viral genomes infected unknown hosts (63%), followed by those predicted to infect Proteobacteria (21%), Cyanobacteria (11%), and Bacteroidetes (2%) (Fig. 2b). This profile is similar to the host prediction of the whole viral community, with the exception of viruses infecting Firmicutes, which were over-represented in the community encoding virulence genes relative to the whole community, and those infecting Actinobacteria, which displayed the opposite pattern (Fig. 2a).

**Fig. 3:**
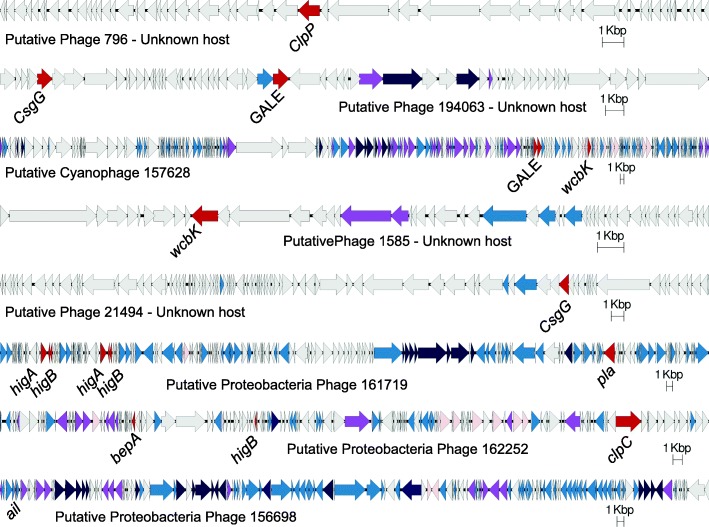
Genomes of predicted viruses encoding bacterial virulence genes. Arrows indicate Open Reading Frames (ORFs) predicted from nucleotide sequences. Bacterial virulence genes are in red, with their specific gene annotation. Gray arrows indicate putative ORFs with unknown function, light blue indicates genes of unknown function identified as phage genes, dark blue indicates phage structural genes, purple indicates an integrase or transposase, and light pink indicates auxiliary metabolic genes. Individual scale bars are provided for each genome

The protein annotations and genome composition of the 30 most abundant viral genomes encoding bacterial virulence genes showed that these genomic sequences varied from 5.4 to 190 Kbp in length and were predicted to infect unknown hosts (13), Proteobacteria (11) and Cyanobacteria (6). Their relative abundances and annotations are provided in Additional file 1: Table S2. About 70% of the open reading frames (ORFs) in these genomes encoded putative proteins with unknown functions, a common characteristic of phage genomes (Fig. 3). The most abundant one, VGS 798 (0.17% of recruited reads), infects an unknown host and except for the predicted virulence gene, all the remaining ORFs encoded putative proteins of unknown function. VGS 194063, the second most abundant, encoded phage structural and replication proteins, and two virulence factors: *csgG* (Curli production assembly/transport component) and UDP-glucose epimerase (GALE). They are followed by Cyanophage VGS 157628, which had a genome 190 Kbp-long, encoded multiple T4-like structural and replication proteins and the genes GALE and *wcbK* (GDP-mannose 4,6-dehydratase). Three Proteobacteria-infecting phage genomes are shown in Fig. 3, two of which encoded *hig* genes, involved in a toxin-antitoxin system used by phages to regulate bacterial protein translation modulating virulence [29]. These proteobacterial phages also encoded virulence genes directly involved in attachment and invasion of eukaryotic hosts: *pla* (Plasminogen activator), *bepA* (Protein adenylyltransferase) and *ail* (attachment and invasion locus).

**Fig. 4:**
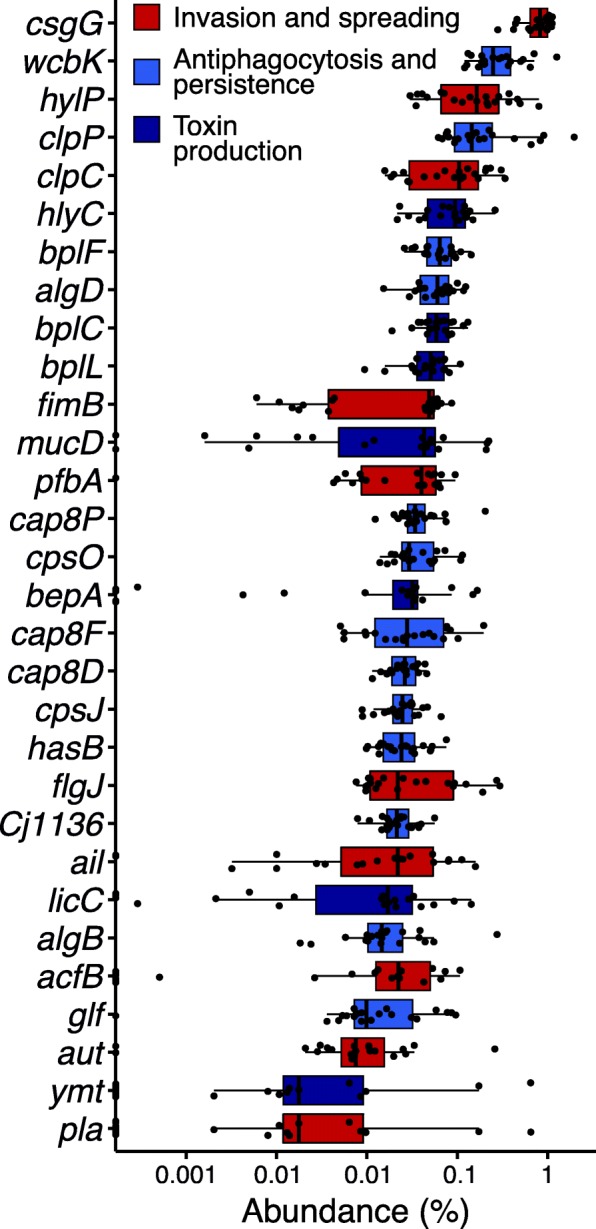
Abundant phage-encoded bacterial virulence genes. The relative abundance of each gene was calculated as the sum of all Viral Genomic Sequences encoding a unique gene. Each dot indicates a virome. The color code is based on broad functions: invasion and spreading, antiphagocytosis and persistence, and toxin production

When summing the abundance of all phage genomic sequences encoding a unique virulence gene, the most abundant genes were involved in eukaryotic host attachment, invasion, immune system evasion, and toxin production (Fig. 4). The most abundant genes were *csgG* (Curli production assembly/transport component, involved in host invasion), *wcbK* (GDP-mannose 4,6-dehydratase, involved in immune evasion), *hylP* (hyaluronidase, involved in spreading through animal tissue), *clpP* and *clpB* (proteases involved in immune system evasion), *hlyC* (hemolysin C, a toxin), and *bplF*, *C* and *L* (Lipopolysaccharide biosynthesis protein, involved in antiphagocytosis), among others. The abundances of the top 30 virulence genes, as calculated by the sum of abundances of all viral genomes encoding a unique gene are provided in Additional file 1: Table S3).

### Drivers of virulence gene profiles

**Fig. 5:**
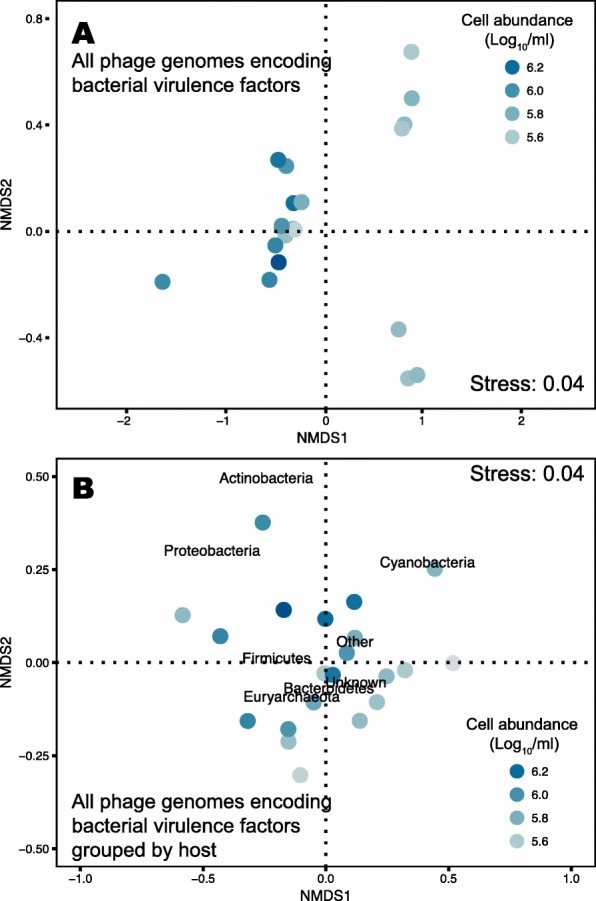
Drivers of phage-encoded bacterial virulence gene profiles. nMDS analyses of **a** microbial abundances and **b** putative hosts as predictors of the relative abundances of viral genomes encoding bacterial virulence genes. Each virome is represented by a circle in the plot color-coded by the microbial abundance (Log10) in that reef site. The distances between the circles represent a two-dimensional reduction of the multi-dimensional analysis of pairwise distances calculated using Bray-Curtis dissimilarities. Permutational linear model tests showed that microbial abundance (A) was a significant predictor of virulence gene profiles (*p* = 0.001), while host was only significant at 90% confidence (*p* = 0.052)

The abundances of viral genomes encoding virulence genes were significantly predicted by environmental microbial abundances (Fig. 5a; permutational linear model *p* = 0.001, pseudo-F_1,19_ = 4.48 using Log_10_ of cell abundance as predictor variable). A second nMDS analysis using the relative abundance of each virulence gene (calculated the sum of all viral genomes encoding that given gene) and cell abundance as predictor showed the same pattern, with virulence gene profile being significantly predicted by cell abundance (Additional file 1: Figure S4, permutational linear model *p* = 0.001, pseudo-F_1,19_ = 4.23 using Log_10_ of cell abundance as predictor variable). Viral genomes were then grouped according to host phylum and host annotation was tested as a predictor of the relative abundances of genomes encoding bacterial virulence genes. This analysis showed that host profile was a weak predictor of virulence gene profiles (Fig. 5b, permutational linear model *p* = 0.052, pseudo-F_1,19_ = 3.14).

**Fig. 6:**
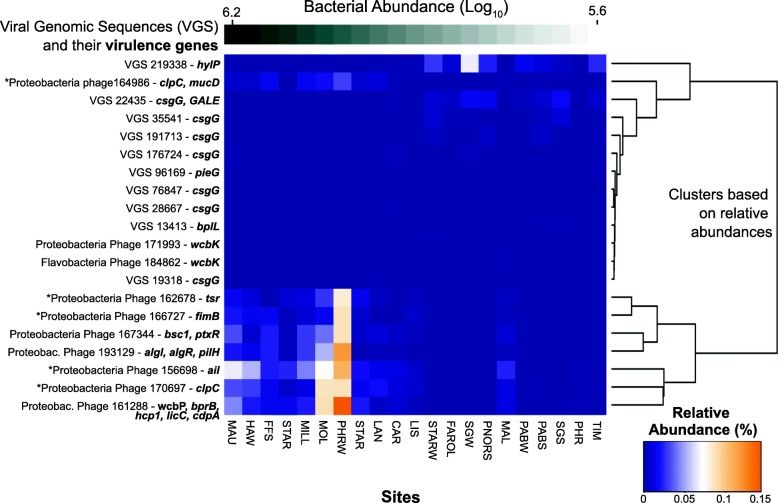
Viruses encoding bacterial virulence genes across the bacterial density gradient. The top 20 Viral Genomic Sequences (VGS) with highest relevance as predictors of cell density, defined by their mean increase accuracy score and significance values (*p* < 0.05) in the permutational regression random forest. The bar at the top depicts the gradient in microbial abundance (Log_10_). The columns indicate each site, ordered by their microbial abundances. VGS are represented in the rows. On the left side, names include VGS unique ID, predicted host, and virulence gene. The asterisk indicates the presence of an integrase of transposase. The cluster on the right side was built based on relative abundances of VGS in each virome

A permutational random forest statistical learning approach determined which virulence gene-encoding genomes were best at predicting the differences across the cell abundance gradient. The random forest analysis showed that the abundance of virulence-encoding genomes explained 39.2% of the variance in cell abundances across viromes. The genomes that displayed high importance on the random forest (increase in mean square error and *p*-values below 0.05 in the permutation) were selected (Fig. 6 and Additional file 1: Figure S5). At high cell abundances, 8 genomes encoding genes involved in two broad functions were enriched: invasion and immune system evasion. The specific genes enriched were *tsr* (chemotaxis and invasion), *fimB* (regulating fimbria assembly for attachment), *ail* (attachment and invasion), and *clp*, *bsc*, *alg* and *muc*, involved in antiphagocytosis. All the eight virulence-encoding VGS enriched at high cell abundance were predicted to infect Proteobacteria, and five encoded an integrase or transposase.

At low microbial abundances, the 12 viral genomes with highest importance in the random forest analysis had lower relative abundances compared to the ones at high microbial abundances (Fig. 6). Ten of these genomes were predicted to infect unknown hosts, one was predicted to infect Proteobacteria and one to infect Flavobacteria. None of these encoded an integrase or transposase. When the gene abundance (as the sum of all phages encoding a unique gene) was tested by the same random forest analysis to predict cell abundance, only 5.06% of the variance was explained (Additional file 1: Figure S6).

## Discussion

### Drivers of phage-encoded virulence gene profiles

Here we tested the hypothesis that in coral reefs, the distribution of phage genes with homology to bacterial virulence factors is associated with microbial densities and host taxonomy. This association is predicted to result from an increased frequency of viral infection and selection of genes that bring competitive advantages to the bacterial host. The results corroborated the first predictions of this hypothesis (the relationship between phage-encoded virulence and microbial density), but did not support the second prediction (relationship between bacterial host and phage virulence genes). The decoupling between functional genes and taxonomy is a common feature of microbial communities and has been previously observed in coral reef microbiomes [25].

The significant relationships between microbial density and the abundance profiles of the whole viral community (Additional file 1: Figure S2) and the fraction of the community encoding virulence factors (Fig. 5a) indicated that host availability is a major driver of phage community structure. These results were consistent with previous observations of viral and bacterial community structure being associated with bacterial densities [19, 23, 27]. The decrease in diversity and richness of virulence-encoding phage genomes with increasing microbial density (Additional file 1: Figure S3) supports the idea of increased abundance of opportunistic strains at high densities [23, 27]. If the acquisition of a virulence gene by a bacterium during lysogeny increases fitness, it would also increase the abundance of this strain in the environment. In this case, high microbial density is an outcome of the gene acquisition, closing a positive feedback loop of microbial biomass accumulation [20, 21].

Phages infecting Proteobacteria were the most abundant among viral genomes for which putative hosts were identified (Fig. 2). Proteobacteria, mainly belonging to the genus *Vibrio*, are common marine pathogens found in high abundances in microbialized reefs, stressed corals, and other animals [25, 28, 30]. Lysogenic conversion was proposed as a virulence mechanism in the coral pathogen *Vibrio corallilyticus*, based on sequence similarity between *V. corallillyticus* prophages and virulence-encoding *V. cholerae* phages [16]. The results described here support the role of lysogenic conversion in coral reef *Vibrio* and extend that to other bacterial groups, suggesting that the lysogenic conversion mechanism is widespread among marine pathogens. Another possible explanation is that these genes are participating in the mediation of commensal or even mutualistic relationships, as marine *Vibrio* can establish diverse symbiotic interactions with eukaryotes [31]. Most virulence-encoding viruses described here infected unknown hosts (Fig. 2), limiting further interpretation of the host-related results, despite the best available tools being applied for host inference [3, 4, 32, 33]. Other biases derived from sample preparation methods could also interfere with these analyses.

### Phage-encoded virulence genes and genomic islands

The most abundant phage-encoded bacterial virulence genes and those enriched at high bacterial densities encoded proteins that are expressed on the bacterial cell surface during phage infection and have functions of invasion, spreading, and immune system evasion (Figs. 4 and 6). The lateral acquisition of these genes and traits is the first step for a bacterial strain that is originally free-living to explore a new niche by associating with an animal host [34, 35]. Exploring this new niche requires successful competition with resident microbiome associated with that animal, and evasion from the animal immune system [36]. Toxins and immune evasion genes perform this function, while other unidentified genes may play roles in bacteria-bacteria competition and bacteria-animal communication. Many of the genes identified here are located in genomic islands or flanked by transposons in reference bacterial genomes. Some examples are: *hlyC*, encoding the toxin hemolysin and found in genomic islands of pathogenic *E. coli* predicted to originate from defective prophages: 10 to 200 kb regions containing an integrase gene, flanked by tRNA genes, and with GC content that significantly deviates from the host genome [37]; Homologs of Clp proteases, some of the most abundant genes in this dataset, are common in bacterial genome and can have different functions, being exchanged between strains through homologous flanking regions. The viral version of this gene is involved in both virion assembly and regulation of the expression of proteins mediating bacterial evasion of immune cells [38, 39]; the genes *csg* and *fim*, involved in the synthesis of two types of fimbria, are enriched at high cell densities and found in genomic islands of bacterial genomes with evidence of horizontal transfer [40]. Fimbria mediate bacterial recognition and invasion of animal hosts, being common in *Pseudovibrio* spp. genomes infecting sponges, corals, flatworms, and tunicates [40].

Virulence genes, specially secreted proteins (toxins) and secretion systems are commonly flanked by mobile genetic elements in bacterial genomes, and transferred along addiction systems, which kill cells in the absence of the mobile genetic element [41]. These genomic islands often originate from integrated phages [42]. Bacterial mutation rates and the selective advantages brought by the virulence gene to the bacteria lead to the complete inactivation of the integrated phage’s ability to form new particles and lyse the host, while maintaining the virulence gene functionality [43].

### Lysogenic conversion in coral reefs

In coral reefs, high rates of lysogenic conversion during microbialization are predicted to contribute to the rise of pathogens [23, 44]. The functional analyses of virulence genes in phage genomes showed that the pathogenic functions encoded by phages enable bacteria to recognize and invade animals. These results suggest that phages are agents of diseases in microbialized systems. The abundant *ail* gene described here (also known as *lom –* lambda outer membrane) is carried by phages infecting *Enterobacteria *spp. and expressed on the outer membrane, triggering attachment to animal epithelial cells and initiation of infection in mice [45]. It is possible that this gene has a similar function in the marine environment, enabling *Enterobacteria* to attach to animal epithelium and explaining the implication of *Enterobacteria* spp. in white-plague disease in the coral species *Orbicella faveolata* and *Mussismilia braziliensis* [46–48]. These predictions provide a platform in the search for potential phage-mediated mechanisms causing marine diseases of unknown pathogen, such as black band disease, white plague, white pox, and stony coral tissue loss [47, 49, 50].

Many genes identified in this in silico analysis as virulence factors may have other functions in vivo. Secreted proteins and secretions systems have been associated with bacteria-bacteria communication and cooperation [41]. Many canonical virulence genes may also enable bacteria to escape protist grazing by modifying bacterial surface, inhibiting their degradation and increasing long-term persistence in association with the eukaryote [51, 52]. The gene *wcbK*, for instance, has immune system evasion roles in Proteobacteria through the modification of cell surface [53, 54], however its presence in Cyanophage genomes may indicate a function in protection against grazing [55, 56]. Here, many of the Cyanophage genomes containing virulence genes did not encode an integrase, preventing the identification of these as lytic or temperate (Fig. 1). However, even in the absence of integration, the expression of phage genes during long-term associations (such as pseudo-lysogeny or simply delayed lysis) may modify bacterial phenotype before death by lysis [57, 58]. Phage T4, for instance, can establish pseudolysogenic infections in *E. coli* through the expression of rI [59]. If that is the case for the Cyanophages found here, lysis delay combined with grazing protection could increase short-term bacterial survival and long term-coexistence of the bacteria and the phage. The combined effects of high virulence and escape from viral and protist predation are predicted to exacerbate coral reef decline and microbialization by increasing bacterial fitness.

## Conclusion

Our study shows that coral reef microbialization is associated with the rise in phage-encoded virulence genes that enable bacterial recognition and invasion of metazoans. The virulence genes encoded by phage genomes were distinct between high and low microbial abundance reefs, suggesting that phage-encoded genes contribute to bacterial fitness at high microbial densities. Predicted bacterial host was not a significant variable driving the virulence gene profiles, indicating that total bacterial density is a stronger predictor of phage-mediated pathogenicity. These results point towards lysogenic conversion as a cause of polymicrobial diseases contributing to coral reef decline.

## Methods

### Viral metagenomic dataset

Viral metagenomes (viromes) from coral reefs analyzed here were previously published in Knowles et al. 2016 [23]. Briefly, samples were collected from reef boundary layers across the Abrolhos Bank in the Southwest Atlantic (7 viromes), the Line Islands archipelago in the central Pacific (6 viromes) and the Hawaiian archipelago (8 viromes). Samples were taken from the benthos-water interface, within 30 cm above the benthic surface [60]. Before library preparation for sequencing, viral DNA was tested for the presence of bacterial 16S by PCR, and only samples that tested negative were analyzed. A description of the 21 viromes analyzed here including site geographical coordinates is provided in Additional file 1: Table S1. Viral and microbial abundances from each sample were determined by epifluorescence microscopy and flow cytometry [23]. 

### Viral genome prediction

A database of viral genomic sequences (VGS) was compiled including i) viral genomes from RefSeq and ii) uncultured marine viral genomes assembled from multiple studies (including the coral reef viromes analyzed here) and amounted to a total of 195,698 sequences [3, 4, 33, 62–66]. The selection of metagenome-assembled viral genomic sequences (VGS) and exclusion of potential bacterial contamination was performed as follows: contigs derived from the assemblies were combined, and those shorter than 2500 bp were removed to decrease the chances of false-positives. Coding DNA sequences were identified with Prodigal within Prokka and protein sequences were queried against the NCBI-nr database for annotation using Diamond, setting a minimum e-value of 10^− 5^. A database of known phage genomes was built by merging genomes obtained from three sources: (1) the NCBI RefSeq database; (2) the complete marine phage genomes obtained from fosmid libraries [66] and (3) prophages identified in bacterial genomes with VirSorter [67]. The database was made non-redundant by clustering the genomes with BLASTn with a 95% identity and a 40% coverage cutoff. The Dice coefficient score was used to estimate the distances between the contigs and the database of known viral genomes to organize them into a phylogenomic framework [66]. Only contigs that had at least one detectable homolog among known viruses as determined by tBLASTx searches were used for further analysis. Reads from the coral reef viral metagenomes were queried against the viral genome database using the very-sensitive-local mode of Bowtie2 [68]. This resulted in 28,483 contigs recruiting at least 10 reads from a given sample. These contigs were selected for visualization with Anvi'o [69]. The top 14,000 contigs in abundance were analyzed with Phanotate [70] to identify ORFs that were compared through BLASTp with a viral RefSeq integrase database.

Microbial hosts infected by VGSs were predicted using multiple bioinformatics methods introduced in Coutinho et al. 2017. Briefly, host prediction was based on: 1) homology matches against bacterial and archaeal genomes; 2) homology matches of the VGSs against the TARA oceans microbial contigs [71]; 3) CRISPR spacers within the microbial genomes [72]; and 4) transfer RNAs identified in VGSs.

### Virulence gene assignment

Coding sequences from VGS were compared to the virulence factors of pathogenic bacteria database from the Pathosystems Resource Integration Center, PATRIC, which contains genes with virulence functions demonstrated in vivo [73]. To avoid false-positives, the PATRIC database sequences were clustered in orthologous groups using the OrthoMCL algorithm within ght GetHomologues pipeline (default parameters, inflation factor = 1.5) [74, 75] and genes encoding ABC transporters, integrases, and chaperones were removed, as these proteins are common in the marine environment and are involved in functions unrelated to virulence. The final curated database is provided the FigShare repository (10.6084/m9.figshare.8232935). Viral proteins predicted from contig ORFs were queried against the curated database using BLASTp with an e-value cut-off of 0.00001 and 40% identity across 20 amino acids. The statistical analyses were performed with either the relative abundances of VGS which encode virulence factors or the abundance of virulence genes, calculated as the sum of the abundances of all VGS in which unique genes were encoded.

### Statistical analyses

Non-metric Multi-Dimensional Scaling (nMDS) followed by permutational multivariate analysis of variance using distance matrices (Adonis) with Bray-Curtis dissimilarity and 999 permutations were performed to test the relationship between bacterial density and host taxonomy. Pairwise distances between samples was calculated from i) the relative abundances of VGS, ii) relative abundance of virulence-encoding VGS, iii) virulence genes as the sum all VGS encoding each gene, and iv) VGS grouped by the host they infect. The analyses were performed using the package Vegan in R [76]. A dimension-reduction analysis was performed using supervised regression random forests in the R package rfPermute with 1000 trees [77].

## Supplementary information

**Additional file 1: Figure S1.** Rank-abundance curve. **Figure S2.** nMDS analysis of the relative abundances of all Viral Genomic Sequences in each virome. **Figure S3.** Relationships between viral community diversity and microbial abundance. **Figure S4.** nMDS analysis of the relative abundances of virulence genes (calculated as the sum of Viral Genomic Sequences encoding that gene in a given sample). **Figure S5.** Viral Genomic Sequences with highest importance in the random forest analysis of the relative abundance of virulence-encoding VGS predicted by the cell abundance gradient. **Figure S6.** Virulence genes with highest importance in the random forest analysis of the relative abundance of virulence genes (as the sum of all phages encoding a given gene) predicted by the cell abundance gradient. **Table S1.** Coral reef virome sampling sites and diversity. **Table S2.** Top 30 most abundant viral genomes encoding virulence genes. **Table S3.** Top 30 most abundant virulence genes across all sites.

## Data Availability

All viromes analyzed here are available on the FigShare repository (10.6084/m9.figshare.4290056.v1 and 10.6084/m9.figshare.4290044.v1). The curated virulence factors database is available on the FigShare repository (10.6084/m9.figshare.8232935).
