# Supplementary material for: Genomic and ecological attributes of marine bacteriophages encoding bacterial virulence genes
Source: BMC Genomics. 2020 Feb 5;21:126. doi: 10.1186/s12864-020-6523-2 (PMC7003362; doi:10.1186/s12864-020-6523-2)
Supplement: Supplementary file 1 — Additional file 1: Figure S1. Rank-abundance curve. Figure S2. nMDS analysis of the relative abundances of all Viral Genomic Sequences in each virome. Figure S3. Relationships between viral community diversity and microbial abundance. Figure S4. nMDS analysis of the relative abundances of virulence genes (calculated as the sum of Viral Genomic Sequences encoding that gene in a given sample). Figure S5. Viral Genomic Sequences with highest importance in the random forest analysis of the relative abundance of virulence-encoding VGS predicted by the cell abundance gradient. Figure S6. Virulence genes with highest importance in the random forest analysis of the relative abundance of virulence genes (as the sum of all phages encoding a given gene) predicted by the cell abundance gradient. Table S1. Coral reef virome sampling sites and diversity. Table S2. Top 30 most abundant viral genomes encoding virulence genes. Table S3. Top 30 most abundant virulence genes across all sites. [file 12864_2020_6523_MOESM1_ESM.docx]

Genomic and ecological attributes of marine bacteriophages encoding bacterial virulence genes

**Silveira et al 2019**

**Supplemental Figures and Tables**

**
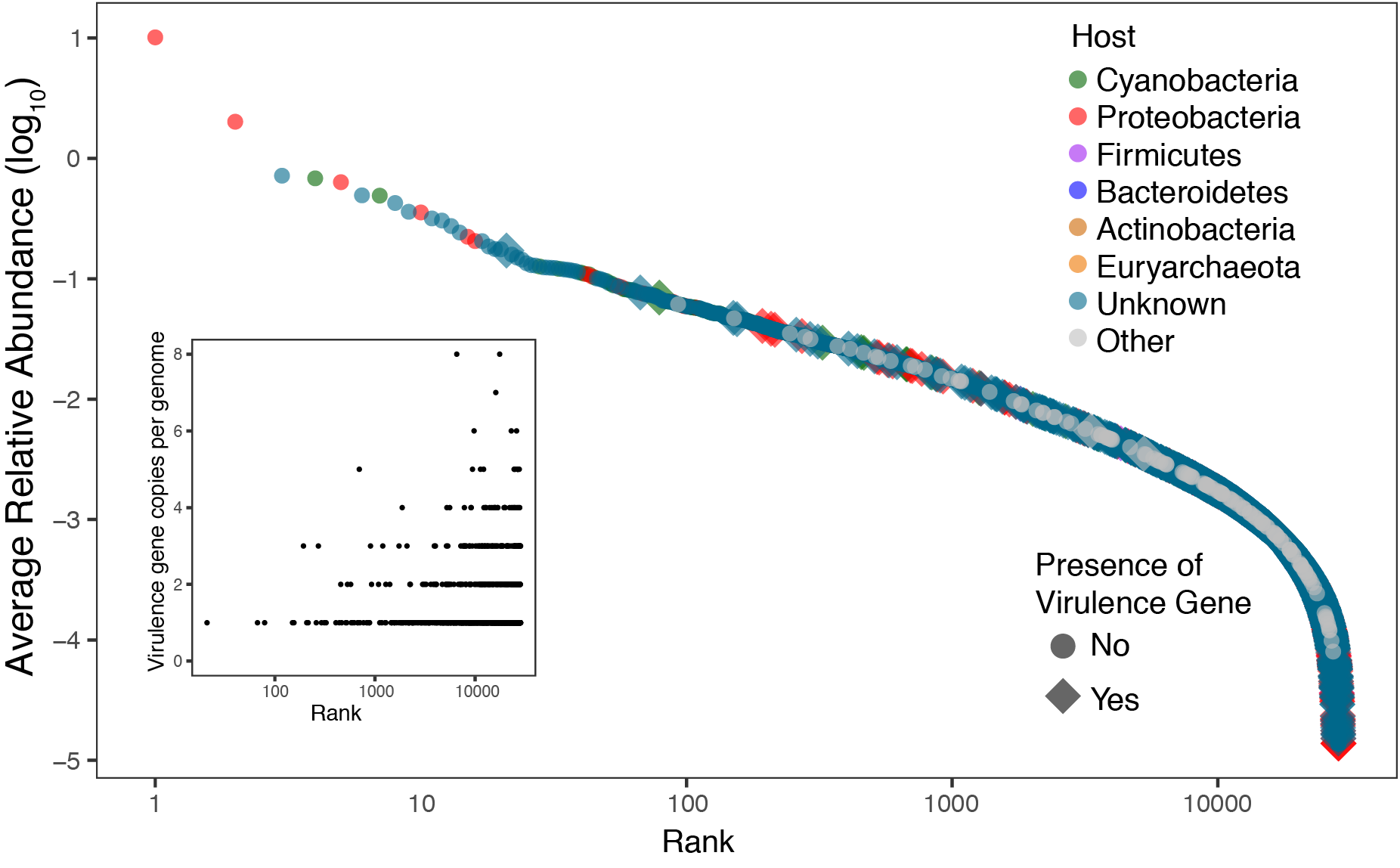
Figure S1. Rank-abundance curve.** The curve was plotted using the mean abundance of each Viral Genomic Sequence (VGS) across all samples. The colors indicate the predicted host, while the shapes indicate the presence of absence of virulence genes. The inlet shows the relationship between phage rank and the number of virulence genes encoded in its genome (linear regression p = 0.08, a = 0.14).

**Figure S2.** nMDS analysis of the relative abundances of all Viral Genomic Sequences in each virome. Virome are color-coded by the microbial abundance (Log_10_) in the sample. Permutational tests showed that microbial abundance was a significant predictor of virulence gene profiles (p = 0.001).

** Figure S3.** Relationships between viral community diversity and microbial abundance. A) Evenness (Shannon index/Species count) calculated from the relative abundance of viral genomes in viromes plotted against microbial abundance (Linear regression p = 0.63). B) Shannon index plotted against microbial abundance (Linear regression p = 0.04). C) Species count versus microbial abundance (Linear regression p = 4.53e-05).

**Figure S4.** nMDS analysis of the relative abundances of virulence genes (calculated as the sum of Viral Genomic Sequences encoding that gene in a given sample). Viromes are color-coded by the microbial abundance (Log_10_) in the sample. Permutational tests showed that microbial abundance was a significant predictor of virulence gene profiles (p = 0.001).

**
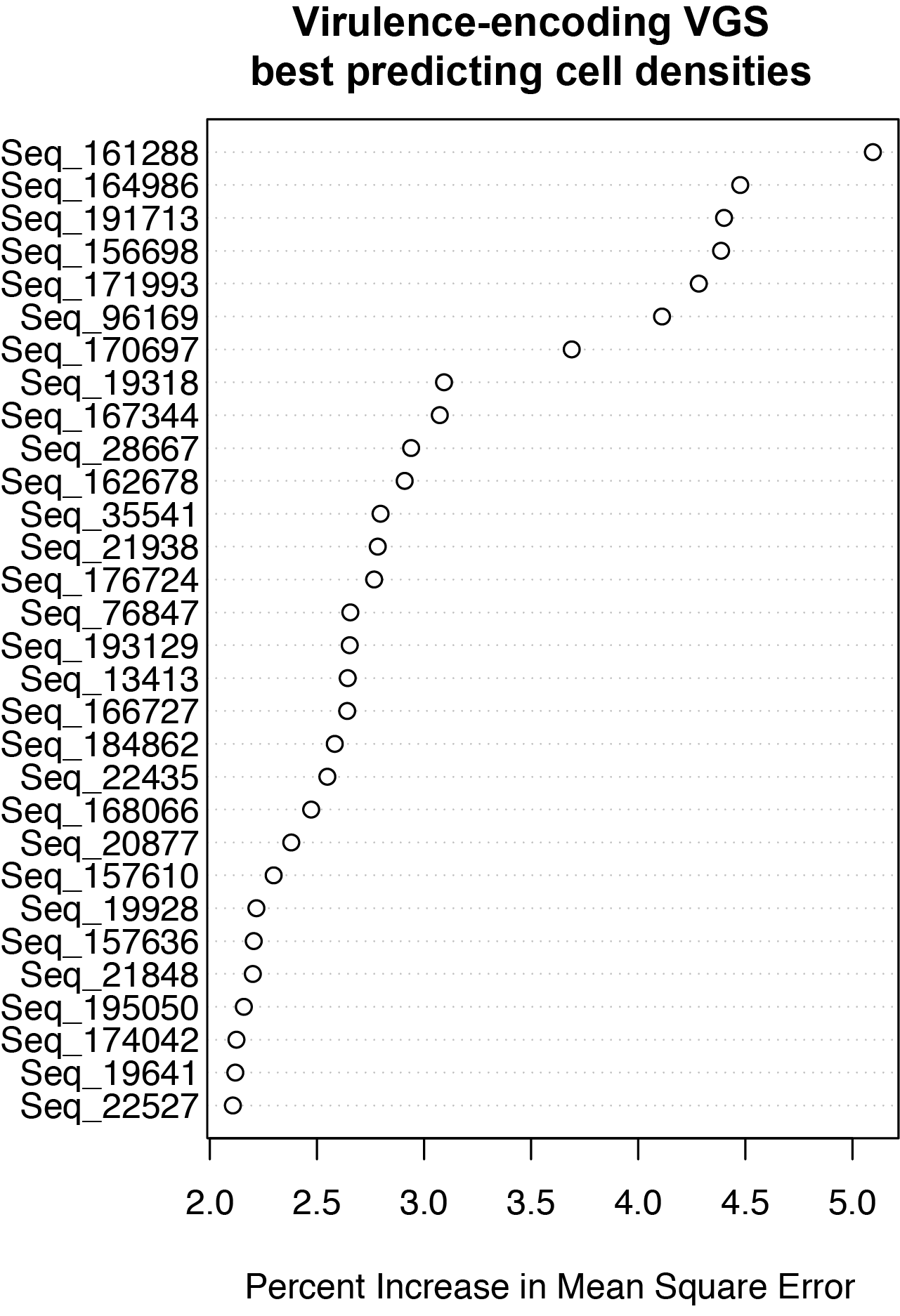
**

**Figure S5.** Viral Genomic Sequences with highest importance in the random forest analysis of the relative abundance of virulence-encoding VGS predicted by the cell abundance gradient. The VGS are ordered according to their importance described by the Percent Increase in Mean Square Error in the random forest. Cell abundance described 39.19 % of the variance in VGS relative abundances.


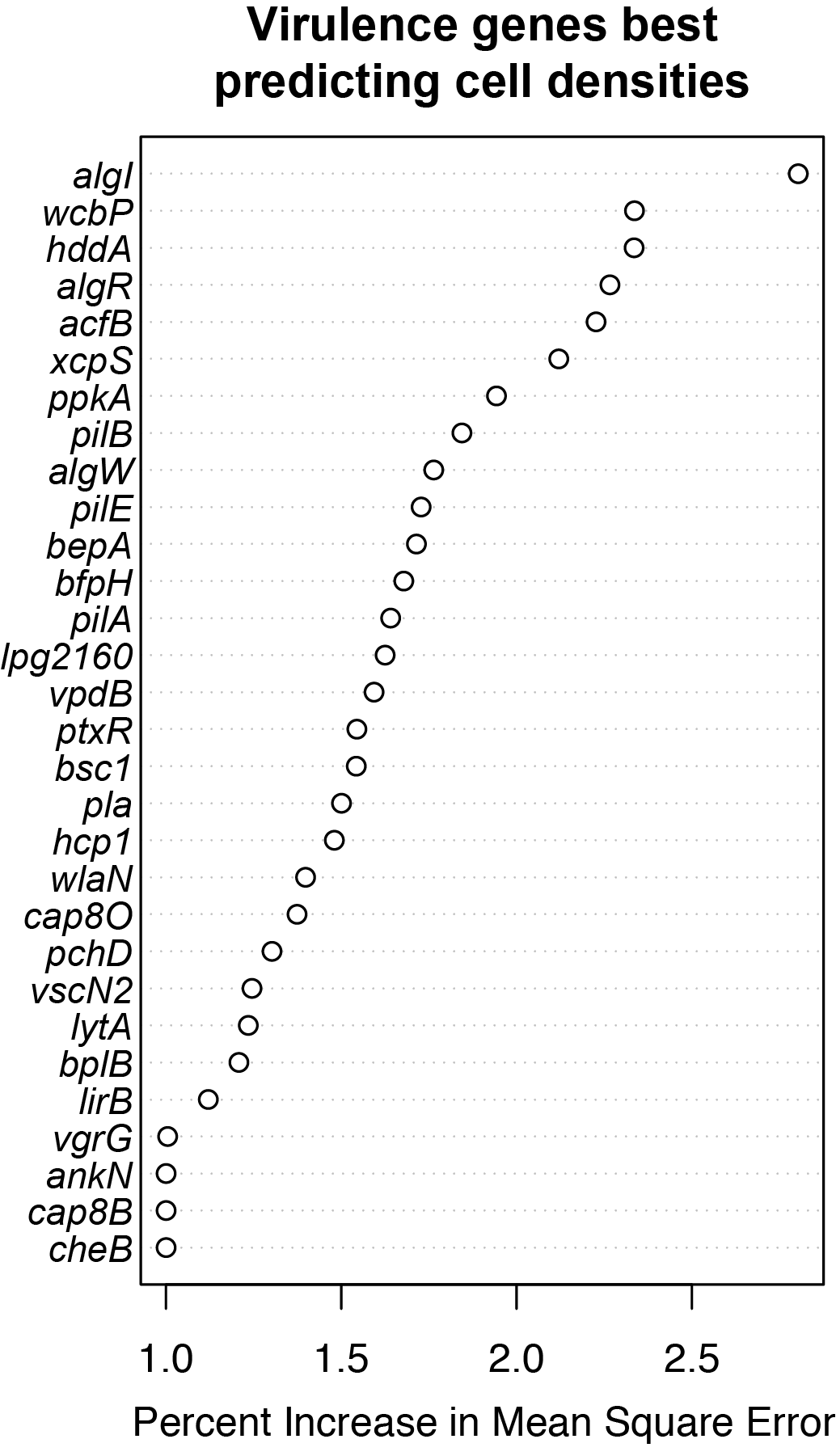


**Figure S6.** Virulence genes with highest importance in the random forest analysis of the relative abundance of virulence genes (as the sum of all phages encoding a given gene) predicted by the cell abundance gradient. The genes are ordered according to their importance described by the Percent Increase in Mean Square Error in the random forest. Cell abundance described 5.06 % of the variance in VGS relative abundances.

**Table S1.** Coral reef virome sampling sites and diversity. Viromes were first published in Knowles, Silveira, et al., 2016.

| Site | Geographic Region | Latitude | Longitude | Cell density  (cells/ml) | Diversity  (H’) | Evenness | Richness | Number of reads |
| --- | --- | --- | --- | --- | --- | --- | --- | --- |
| Millenium atoll (Car09) | Line Islands, Central Pacific | 9.91672 | -150.21072 | 736330 | 7.92753335 | 0.83423909 | 13396 | 259736 |
| French Frigate Shoals | Northwestern Hawaiian Islands | 23.87748 | -166.29115 | 1513134 | 7.14384682 | 0.84000656 | 4937 | 27836 |
| Farol | Abrolhos, Western Atlantic | -17.965 | -38.694 | 546163 | 6.8331546 | 0.66931303 | 27152 | 5001717 |
| Hawaii | Main Hawaiian Islands | 20.00324 | -155.83318 | 1586834 | 7.4638355 | 0.81343066 | 9660 | 169868 |
| Lanai | Main Hawaiian Islands | 20.76167 | -156.8334 | 918810 | 7.76916372 | 0.833401 | 11184 | 216812 |
| Lisianksi | Northwestern Hawaiian Islands | 25.98702 | -173.99438 | 641006 | 7.9545931 | 0.83994318 | 12970 | 197911 |
| Maui | Main Hawaiian Islands | 20.86447 | -156.13911 | 2177100 | 7.18152785 | 0.77934456 | 10045 | 210777 |
| Molokai | Main Hawaiian Islands | 21.17481 | -156.8214 | 1137824 | 6.39275273 | 0.7097056 | 8165 | 131396 |
| Malden | Line Islands, Central Pacific | 3.99531 | -154.94452 | 492594 | 7.68203958 | 0.81240948 | 12783 | 273811 |
| Millennium atoll | Line Islands, Central Pacific | 9.90774 | -150.19974 | 1151554 | 7.05993431 | 0.76576122 | 10092 | 173813 |
| Parcel dos Abrolhos (w) | Abrolhos, Western Atlantic | -17.983644 | -38.667725 | 468294 | 7.29691408 | 0.73905628 | 19405 | 869784 |
| Parcel dos Abrolhos (s) | Abrolhos, Western Atlantic | -17.983644 | -38.667725 | 410856 | 8.35098507 | 0.81781777 | 27209 | 3264729 |
| French Frigate Shoals (54) | Northwestern Hawaiian Islands | 27.79316 | -175.99635 | 327329 | 7.98781758 | 0.81616058 | 17802 | 897002 |
| French Frigate Shoals (w) | Northwestern Hawaiian Islands | 27.86674 | -175.73599 | 883319 | 4.53766955 | 0.5775821 | 2582 | 88980 |
| Portinho Norte | Abrolhos, Western Atlantic | -17.96 | -38.701828 | 519290 | 7.84302597 | 0.78864667 | 20846 | 717677 |
| Sebastiao Gomes (s) | Abrolhos, Western Atlantic | -17.913969 | -39.145803 | 540410 | 7.35871715 | 0.76982461 | 14171 | 317378 |
| Sebastiao Gomes (w) | Abrolhos, Western Atlantic | -17.913969 | -39.145803 | 403270 | 8.52820901 | 0.85148791 | 22374 | 1076752 |
| Santa Barbara | Abrolhos, Western Atlantic | -17.964778 | -38.702778 | 585775 | 8.89048009 | 0.8985323 | 19820 | 323551 |
| Starbuck (7) | Line Islands, Central Pacific | 5.6222 | -155.88002 | 956273 | 7.67353972 | 0.820363 | 11543 | 209465 |
| Starbuck | Line Islands, Central Pacific | 5.66441 | -155.87346 | 1431753 | 7.38638003 | 0.8097955 | 9148 | 158078 |
| Timbebas | Abrolhos, Western Atlantic | -17.482108 | -39.013414 | 262052 | 7.7325537 | 0.776611 | 21095 | 932966 |

**Table S2. Top 30 most abundant viral genomes encoding virulence genes.** The first column indicates the ID of the viral genome in the dataset, the second column indicates the rank based on the mean percent abundance of this genome across all sites (column 5); the third column indicates the size of the genomic sequence in base pairs. The Virulence Factor database ID refers to the unique identification number of a virulence factor in our curated database; This ID is followed by its corresponding GenBank ID, gene name and virulence function.

| **Viral Genomic Sequence** | **Rank** | **Genome size (bp)** | **Host Phylum** | **Mean Abundance (%)** | **Virulence Factor database ID** | **VF Gene Bank accession** | **VF gene name** | **VF Function** |
| --- | --- | --- | --- | --- | --- | --- | --- | --- |
| VGS_796 | 21 | 32843 | Unknown | 0.1714 | VFG000077 | NP_465991 | *clpP* | Protease |
| VGS_194063 | 67 | 38682 | Unknown | 0.0768 | VFG000462 | NP_460110 | *csgG* | Adherence; Fimbrial |
| VGS_157628 | 79 | 190789 | Cyanobacteria | 0.0697 | VFG002556 | YP_109392 | *wcbK* | Antiphagocytosis |
| VGS_1585 | 150 | 26804 | Unknown | 0.0472 | VFG002556 | YP_109392 | *wcbK* | Antiphagocytosis |
| VGS_21949 | 155 | 55844 | Unknown | 0.0461 | VFG000462 | NP_460110 | *csgG* | Adherence; Fimbrial |
| VGS_161719 | 193 | 114570 | Proteobacteria | 0.0405 | VFG000422, VFG000417, VFG014950, VFG000079 | NP_395420, NP_395233, NP_395233 | *ymt, pla* | Toxin |
| VGS_21783 | 208 | 30709 | Proteobacteria | 0.0384 | VFG000905 | NP_755444 | *hlyC* | Toxin; Membrane-damaging; Pore-forming; RTX toxin |
| VGS_190773 | 215 | 42321 | Proteobacteria | 0.0376 | VFG000462 | NP_460110 | *csgG* | Adherence; Fimbrial |
| VGS_86303 | 259 | 16978 | Unknown | 0.0343 | VFG000462 | NP_460110 | *csgG* | Adherence; Fimbrial |
| VGS_162252 | 272 | 76308 | Proteobacteria | 0.0334 | VFG002241, VFG014950, VFG000079 | YP_034062, NP_249457, NP_463763 | *bepA, mucD, clpC* | Secretion system; Type IV secretion system; Protease |
| VGS_21954 | 292 | 51560 | Unknown | 0.0316 | VFG000462 | NP_460110 | *csgG* | Adherence; Fimbrial |
| VGS_190050 | 312 | 17935 | Unknown | 0.0300 | VFG000077 | NP_465991 | *clpP* | Protease |
| VGS_1937 | 325 | 15054 | Cyanobacteria | 0.0294 | VFG001302 | NP_644944 | *cap8F* | Antiphagocytosis |
| VGS_187040 | 407 | 33065 | Unknown | 0.0262 | VFG000905 | NP_755444 | *hlyC* | Toxin; Membrane-damaging; Pore-forming; RTX toxin |
| VGS_13429 | 440 | 6845 | Unknown | 0.0250 | VFG000871 | NP_757239 | *fimB* | Adherence; Invasion |
| VGS_157620 | 455 | 175430 | Cyanobacteria | 0.0245 | VFG002343, VFG000965 | YP_001006756, NP_268936 | *flgJ, hylP* | Secretion system; Invasion; Motility; Exoenzyme; Spreading factor |
| VGS_157617 | 465 | 171797 | Cyanobacteria | 0.0242 | VFG000965 | NP_268936 | *hylP* | Exoenzyme; Spreading factor |
| VGS_10838 | 510 | 5421 | Unknown | 0.0226 | VFG043465 | NP_359244 | *pfbA* | Adherence; Fibronectin-binding protein |
| VGS_176190 | 522 | 48414 | Proteobacteria | 0.0223 | VFG000462 | NP_460110 | *csgG* | Adherence; Fimbrial |
| VGS_156698 | 531 | 103445 | Proteobacteria | 0.0221 | VFG000354, VFG000029 | NP_406409, NP_230488 | *ail, acfB* | Invasion; Serum resistance; Adherence |
| VGS_190241 | 572 | 81334 | Proteobacteria | 0.0210 | VFG002556 | YP_109392, NP_878986 | *wcbK, bplL* | Antiphagocytosis; Endotoxin |
| VGS_82803 | 579 | 13144 | Unknown | 0.0209 | VFG000462 | NP_460110 | *csgG* | Adherence; Fimbrial |
| VGS_161826 | 597 | 77652 | Proteobacteria | 0.0205 | VFG000116 | NP_254170 | *algB* | Antiphagocytosis; Serum resistance; Endotoxin |
| VGS_191091 | 620 | 13714 | Unknown | 0.0202 | VFG000462 | NP_460110 | *csgG* | Adherence; Fimbrial |
| VGS_155993 | 671 | 178249 | Cyanobacteria | 0.0195 | VFG000965 | NP_268936 | *hylP* | Exoenzyme; Spreading factor |
| VGS_157627 | 677 | 174079 | Cyanobacteria | 0.0194 | VFG000965 | NP_268936 | *hylP* | Exoenzyme; Spreading factor |
| VGS_161288 | 697 | 98319 | Proteobacteria | 0.0190 | VFG002440, VFG000328, VFG045467, VFG002550, VFG002071 | YP_111528, NP_439688, YP_107884, YP_109386, NP_248775 | *bprB, licC, cdpA, wcbP, hcp1* | Secretion system; Type III and VI secretion system; Invasion; Signaling; Intracellular signaling; Antiphagocytosis |
| VGS_169837 | 708 | 25933 | Proteobacteria | 0.0188 | VFG000079 | NP_463763 | *clpC* | Protease |
| VGS_170821 | 768 | 59013 | Proteobacteria | 0.0178 | VFG014984 | NP_253136 | *algW* | Adherence; Biofilm formation |
| VGS_4737 | 827 | 14726 | Unknown | 0.0167 | VFG002343 | YP_001006756 | *flgJ* | Secretion system; Invasion; Motility |
|  |  |  |  |  |  |  |  |  |

**Table S3. Top 30 most abundant virulence genes across all sites.** The first column indicates the gene name, followed by its virulence function, corresponding GenBank Access number and the mean percent abundance of this genome across all sites.

| VF gene | Function | GenBank | Mean Abundance (%) |
| --- | --- | --- | --- |
| *csgG* | Protease; Hydrolase; Amidase | NP_465991 | 0.807 |
| *wcbK* | Antiphagocytosis; Adherence; Tissue invasion | NP_460110 | 0.346 |
| *clpP* | Invasion; Serum resistance | YP_109392 | 0.326 |
| *hylP* | Secretion system; Type III secretion system; Invasion | NP_395420 | 0.215 |
| *clpC* | Secretion system; Invasion; Motility | NP_755444 | 0.118 |
| *hlyC* | Invasion | YP_034062 | 0.095 |
| *bplF* | Toxin; Membrane-damaging; RTX toxin | NP_644944 | 0.067 |
| *flgJ* | Antiphagocytosis | NP_757239 | 0.066 |
| *bplC* | Secretion system; Type III secretion system | YP_001006756 | 0.064 |
| *algD* | Exoenzyme; Spreading factor | NP_268936 | 0.063 |
| *mucD* | Toxin; A-B type; N-glycosidase | NP_359244 | 0.054 |
| *bplL* | Endotoxin | NP_406409 | 0.051 |
| *cap8F* | Endotoxin | NP_254170 | 0.045 |
| *cap8P* | Adherence; Twitching motility | YP_111528 | 0.044 |
| *cpsO* | Endotoxin | NP_463763 | 0.042 |
| *pla* | Adherence | NP_253136 | 0.041 |
| *ymt* | Toxin | NP_438708 | 0.041 |
| *fimB* | Adherence; Motility | NP_249453 | 0.036 |
| *pfbA* | Secretion system; Type III secretion system; Invasion | NP_688174 | 0.035 |
| *bepA* | Toxin; RTX toxin | NP_270108 | 0.033 |
| *ail* | Adherence; Invasion | NP_252238 | 0.033 |
| *algB* | Secretion system; Invasion; Motility | AAP42192 | 0.031 |
| *hasB* | Adherence; Type IV pilus | NP_878986 | 0.029 |
| *cpsJ* | Adherence | NP_879577 | 0.027 |
| *cap8D* | Endotoxin | NP_252230 | 0.027 |
| *licC* | Secretion system; Type IV secretion system | NP_644954 | 0.025 |
| *glf* | Secretion system; Type IV secretion system | YP_109900 | 0.024 |
| *Cj1136* | Antiphagocytosis | NP_282580 | 0.024 |
| *acfB* | Secretion system; Type IV secretion system | NP_800848 | 0.022 |
| *aut* | Antiphagocytosis; Adherence; Tissue invasion | NP_345446 | 0.022 |
